# The POLD3 subunit of DNA polymerase δ can promote translesion synthesis independently of DNA polymerase ζ

**DOI:** 10.1093/nar/gkv023

**Published:** 2015-01-27

**Authors:** Kouji Hirota, Kazunori Yoshikiyo, Guillaume Guilbaud, Toshiki Tsurimoto, Junko Murai, Masataka Tsuda, Lara G. Phillips, Takeo Narita, Kana Nishihara, Kaori Kobayashi, Kouich Yamada, Jun Nakamura, Yves Pommier, Alan Lehmann, Julian E. Sale, Shunichi Takeda

**Affiliations:** 1 Department of Radiation Genetics, Graduate School of Medicine, Kyoto University, Yoshidakonoe, Sakyo-ku, Kyoto 606-8501, Japan; 2 Department of Chemistry, GraduateSchool of Science and Engineering, Tokyo Metropolitan University, Minami-Osawa, Hachioji- shi, Tokyo 192-0397, Japan; 3 Medical Research Council Laboratory of Molecular Biology, Hills Road, Cambridge CB2 0QH, UK; 4 Department of Biology, School of Sciences, Kyushu University, 6-10-1 Hakozaki, Higashi-ku, Fukuoka 812-8581, Japan; 5 Laboratory of Molecular Pharmacology, Center for Cancer Research, National Cancer Institute, National Institutes of Health, Bethesda, MD 20892, USA; 6 Division of Genetic Biochemistry, National Institute of Health and Nutrition, Tokyo 162-8636, Japan; 7 Department of Environmental Sciences and Engineering, University of North Carolina, Chapel Hill, NC 27599, USA; 8 Genome Damage and Stability Centre, University of Sussex, Falmer, Brighton BN1 9RQ, UK

## Abstract

The replicative DNA polymerase Polδ consists of a catalytic subunit POLD1/p125 and three regulatory subunits POLD2/p50, POLD3/p66 and POLD4/p12. The ortholog of POLD3 in *Saccharomyces cerevisiae*, Pol32, is required for a significant proportion of spontaneous and UV-induced mutagenesis through its additional role in translesion synthesis (TLS) as a subunit of DNA polymerase ζ. Remarkably, chicken DT40 B lymphocytes deficient in POLD3 are viable and able to replicate undamaged genomic DNA with normal kinetics. Like its counterpart in yeast, POLD3 is required for fully effective TLS, its loss resulting in hypersensitivity to a variety of DNA damaging agents, a diminished ability to maintain replication fork progression after UV irradiation and a significant decrease in abasic site-induced mutagenesis in the immunoglobulin loci. However, these defects appear to be largely independent of Polζ, suggesting that POLD3 makes a significant contribution to TLS independently of Polζ in DT40 cells. Indeed, combining *polη, polζ* and *pold3* mutations results in synthetic lethality. Additionally, we show *in vitro* that POLD3 promotes extension beyond an abasic by the Polδ holoenzyme suggesting that while POLD3 is not required for normal replication, it may help Polδ to complete abasic site bypass independently of canonical TLS polymerases.

## INTRODUCTION

Polδ and Polϵ are the major eukaryotic replicative polymerases. Polδ has been shown to be predominantly responsible for lagging DNA strand synthesis during replication (1) but is also required for base and nucleotide excision repair (2–4), break induced replication and homologous recombination (HR) (5,6). Vertebrate Polδ consists of four subunits: POLD1/p125, the catalytic subunit, POLD2/p50, POLD3/p66 and POLD4/p12, which serve regulatory functions (7,8). Consistent with the central role of Polδ in genome replication and repair, POLD1 and POLD2 are both essential for cell proliferation (9–11). However, while the POLD3 ortholog in the fission yeast *Schizosaccharomyces pombe* is essential (12), the ortholog in the budding yeast *Saccharomyces cerevisiae*, Pol32, is not (11). Pol32-deficient *S. cerevisiae* exhibit defects in Polζ-dependent mutagenesis as well as in double-strand break repair by break-induced DNA replication (5–6,13–15). Recent reports have explained the role of Pol32 in mutagenesis by showing that, along with Pol31 (the yeast ortholog of POLD2) it is an integral component of the specialized translesion synthesis (TLS) polymerase Polζ (16,17). Moreover, POLD2 and POLD3 are common subunits of both Polδ and Polζ in mammalian cells (16–19), interacting with the iron–sulphur clusters found in the C-terminus of both Polδ and Polζ (19). The current model of TLS suggests that when replicative polymerases encounter DNA lesions they hand off DNA synthesis to one of a number of specialized translesion polymerases, which are able to insert nucleotides opposite the lesion. The resulting mismatch is further extended by Polζ, creating a conventional 3′ end from which the replicative polymerases can continue DNA synthesis (20,21). It has been suggested that POLD2 and POLD3, being subunits of both Polδ and Polζ, could contribute to this switch (18) although there is currently no direct evidence for this idea.

The *in vivo* role of POLD3 in vertebrate replication and TLS has not yet been defined. Here we have generated POLD3-deficient cells (*pold3* cells) from the chicken DT40 B lymphocyte line. DT40 cells provide an opportunity for precisely examining *in vivo* TLS across abasic sites by sequencing the constitutively diversifying immunoglobulin light-chain variable gene (IgL). During avian IgL diversification, the action of activation induced deaminase (AID) leads to conversion of dC to dU, which is then efficiently excised by uracil DNA glycosylase to yield a high frequency of abasic sites (22,23), the most common spontaneously-arising lesion in the chromosomal DNA (24), within a defined window of around 500 bp. In DT40 these abasic sites can induce gene conversion with a set of homeologous upstream IgL pseudogenes, resulting in templated mutagenesis or can be bypassed directly by TLS resulting in nontemplated mutagenesis at dC/dG basepairs (22,25–28). Here we show that *pold3* cells replicate with essentially normal kinetics but exhibit a 4.5-fold decrease in the nontemplated immunoglobulin mutagenesis, indicating that POLD3 is required for efficient TLS over abasic sites. Further, *pold3* cells exhibit a marked defect in maintaining replication fork progression following UV irradiation. Neither of these phenotypes is observed in cells lacking both Polη and Polζ, and combined loss of Polη, Polζ and POLD3 results in lethality. Together, these results are consistent with POLD3 playing a critical role in TLS in DT40 cells that is substantially independent of Polζ.

## MATERIALS AND METHODS

### Disruption of POLD3 in DT40 cells

**Figure 1.**
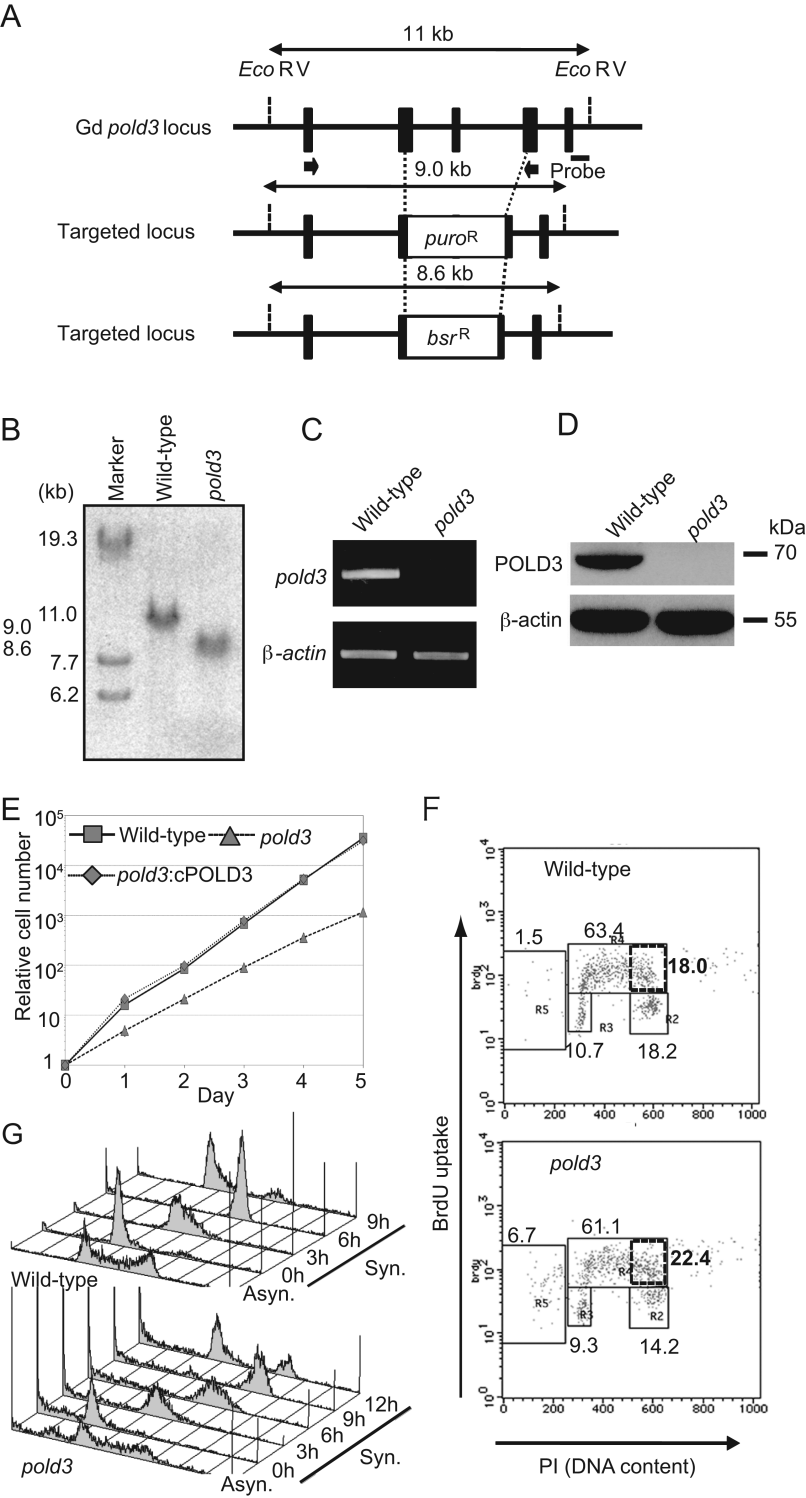
*pold3* cells exhibit a prolonged S-phase. (**A**) *POLD3* disruption in DT40 cells. The wild-type chicken *POLD3* locus from exon 6 to exon 8 was replaced by a *puro* or *bsr* selection-marker gene. Targeted loci (middle and bottom) are shown and compared with the relevant chicken *POLD3* genomic sequences (top). Solid boxes indicate the position of the exons. Relevant EcoRV sites and the position of the probe used in the Southern blot analysis are indicated. Black arrows indicate the position of primers used for RT-PCR in (**C**). (**B**) Disruption of *POLD3* was confirmed by Southern blot. (C and **D**) Depletion of *POLD3* mRNA and POLD3 protein in *pold3* cells was confirmed by RT-PCR (C) and western blot (D). β-actin was used as an internal control. (**E**) Relative growth rate plotted for the indicated genotypes. (**F**) Representative cell-cycle distribution for the indicated genotypes. The top of the box, and the lower left, lower right, and left-most gates correspond to cells in the S, G_1_ and G_2_/M phases, and the sub-G_1_ fraction, respectively. The sub-G_1_ fraction represents dying and dead cells. The percentage of cells in each gate is indicated. The box outlined with bold lines corresponds to cells in the late S phase, with the bolded number indicating the percentage of cells. (**G**) Cells of the indicated genotypes were synchronized at the G_1_ phase with elutriation and released into culture. Cell-cycle progression profiles after release are shown.

Constructs for targeting and disrupting the chicken *POLD3* locus were generated from genomic polymerase chain reaction (PCR) products combined with *puro^R^* and *bsr^R^* selection-marker cassettes (Figure 1A). Genomic DNA sequences were amplified using primers 5′-GGGGCTCGAGCTGCAGCAAGTGAGTTGGTGTTCCTTC-3′ and 5′-GGGGAATTCCTGTGAGCTCTGCTTAGCAGCTGTAGGACC-3′ for the 5′-arm of the targeting construct and 5′-GGGACTAGTGTCAACTCAGTGCCTGGGCAGCCAAAGGAGG-3′ and 5′-GGGGCGGCCGCCCTTCCTCATCACTGCTGTCAGACTCC-3′ for the 3′-arm of the targeting construct. The resulting 3.6 kb 3′-arm and 1.5 kb 5′-arm were cloned into the XhoI–EcoRI and SpeI–NotI sites, respectively, of pBlueScript SK. *puro^R^* and *bsr^R^* selection cassettes, flanked by loxP sequences, were inserted into the BamHI site to generate *pold3*-*puro^R^* and *pold3-bsr^R^* targeting constructs. To generate *pold3* cells, wild-type DT40 cells were transfected sequentially with *pold3-puro^R^* and *pold3-bsr^R^*. Targeted integration of the constructs was detected by Southern blotting of EcoRV- digested genomic DNA and a 0.70 kb probe generated by PCR from the genomic *POLD3* locus with the primers 5′-GGCAGCCACGTGGGTCAGGGGCC-3′ and 5′-CTGGAGCTGTACACTGCACTGTTGG-3′. Targeting efficiency for the first and second allele was 12.5% (5/40) and 2% (2/100) respectively.

### RT-PCR

The loss of *POLD3* transcript was confirmed by RT-PCR using primers 5′-ACTGCAGCAAGTTCAGTGCC-3′ and 5′-CTCTACTATGCTATTAGGAG-3′. β-actin transcripts were analyzed as a positive control for the RT-PCR analysis using primers 5′-CATTGCTGACAGGATGCAGAAGG-3′ and 5′-TGCTTGCTGATCCACATCTGCTGG-3′.

### Construction of POLD3 cDNA-expression vector

Chicken *POLD3* cDNA was isolated by PCR amplification using primers 5′-GACTGAGCGGCCGCATGGAGGACGAGCTGTACCTCGA-3′ and 5′-CTGACTCCAGCACACTGGTCATTTCTTCTGACAGAAGCCCATG-3′ and inserted into a pIRES2–EGFP expression vector (Invitrogen, Carlsbad, CA, USA).

### Assessment of sensitivity of cells to genotoxic agents

Sensitivity to γ-rays, UV light, cisplatin and camptothecin was measured as a fraction of surviving colonies. A colony-formation assay was performed as described previously (29). Briefly, to analyze sensitivity to γ-rays, cells were plated into medium containing methylcellulose and subsequently irradiated with a ^137^Cs γ-ray source. For exposure of cells to UV light, 3 × 10^5^ cells were suspended in 0.5 ml of PBS (phosphate-buffered saline) containing 1% FCS (fetal calf serum) in 6-well plates and irradiated with UVC (254 nm wavelength). For exposure of cells to cisplatin (Nihon-Kayaku, Tokyo, Japan), 1 × 10^5^ cells were treated for 1 h at 39.5°C in 1 ml of complete medium containing cisplatin. Sensitivity to camptothecin, Methyl methansulfonate (MMS) and H_2_O_2_ was measured as a fraction of living cells after proliferation in liquid culture. Cells were exposed to camptothecin continuously. For exposure of cells to MMS or H_2_O_2_, 2 × 10^5^ cells were treated for 1 h at 39.5°C in 1 ml of PBS containing 1% FCS and MMS or complete medium containing H_2_O_2_. Two hundred cells were seeded into 384-well white plates (#6007680 Perkin Elmer Life Sciences, Waltham, MA, USA) with 40 μl of medium per well. Plates were incubated at 39.5°C for 72 h. Cell survival was determined using the ATPlite 1-step kit (PerkinElmer). Briefly, 40 μl ATPlite solution was added to each well. After 5 min, luminescence was measured by Envision 2104 Multilabel Reader (PerkinElmer).

### Flow-cytometric analysis of cell-cycle progression

To analyze cell-cycle progression, 1 × 10^6^ cells were exposed for 10 min to 20 μM 5-bromo-2’-deoxyuridine (BrdUrd; Nacalai Tesque, Kyoto, Japan), then harvested and fixed with 70% ethanol. Fixed cells were incubated with 2 M HCl containing Triton X-100 at 0.5%, treated with mouse anti-BrdUrd monoclonal antibody (BD PharMingen, San Diego, CA, USA) and then with FITC-conjugated anti-mouse IgG antibody (Southern Biotechnology Associates, Birmingham, AL, USA). Cells were resuspended in PBS containing propidium iodide (PI) at 5 μg/ml for subsequent analysis using FACScaliber (BD Biosciences). To measure the progression of the cell cycle, cells were synchronized at the G_1_ phase using centrifugal counterflow elutriation (Hitachi Industrial, Tokyo, Japan). The cell suspension (∼5 × 10^7^ cells) was loaded at a flow rate of 11 ml/min into an elutriation chamber running at 2000 rpm. Cell synchrony was confirmed by Fluorescence-activated cell sorting (FACS) analysis.

### Dynamic molecular combing and immunofluorescent detection

Asynchronously growing DT40 cells were sequentially labelled for 15 min with 25 μM IdU and for 15 min with 25 μM CIdU. UV treated cells were irradiated at 20 J/m^2^ just before the CldU treatment. At the end of the labeling period (30 min), cells were placed in ice cold 1× PBS (1 volume of cells for 2 volumes of 1× PBS) and centrifuged at 250 g for 5 min at 4°C, washed in ice-cold PBS, and resuspended in PBS to a final concentration of 1 × 10^6^ cells/ml. Three microliter of the cell suspension was spotted onto clean glass Superfrost slides and lysed with 7 μl of 0.5% sodium dodecyl sulphate (SDS) in 200 mM Tris–HCl (pH 5.5) and 50 mM ethylenediaminetetraacetic acid (EDTA) (5 min, at room temperature). Slides were tilted at 15° to horizontal, allowing the DNA to run slowly down the slide. Slides were then air dried and fixed in 3:1 methanol/acetic acid and stored at 4°C before immunolabeling. IdU, CldU, DNA revelations and analysis were performed as described (30), with minor modifications: the DNA was denatured for 30 min in 2.5 N HCl, and CldU was detected using rat anti BrdU (ABD Serotec, Raleigh, NC, USA) at 1/750. A stretching factor of 2.6 for conversion from μm to kb was applied, as previously described for the method in (31). Slides were mounted in 10% 1× PBS and 90% glycerol, kept at −20°C and imaged using a Nikon C1-si confocal microscope.

### AID overexpression by retrovirus infection

Wild-type and *pold3* cells were inoculated in 96-well plates to obtain single colonies (Supplementary Figure S4). Several single colonies were picked up and genomic DNA was extracted. After the DNA sequence of the V(D)J locus was analyzed, clones without any mutation or gene conversion in the V(D)J locus were obtained. AID overexpression was carried out by infection of retrovirus containing the *AID* gene and the internal ribosomal entry site (IRES) followed by the Green fluorescent protein (GFP) gene (Supplementary Figure S4), as described previously (27,32). The efficiency of infection was about 70%, as assayed by GFP expression. The per division rate of Ig-gene conversion and hypermutation (events/IgV segment/division) was calculated assuming doubling times of 7.6 and 11 h for wild-type and *pold3* cells respectively.

### Analysis of Ig Vλ diversification

Genomic DNA was extracted at 14 days post infection. Using primers 5′-CAGGAGCTCGCGGGGCCGTCACTGATTGCCG-3′, in the Vλ leader intron and 5′-GCGCAAGCTTCCCCAGCCTGCCGCCAAGTCCAAG-3′, in the J-Cλ intron, the rearranged Vλ segments were PCR amplified, with Prime Star DNA polymerase (Takarabio, Shiga, Japan), a high fidelity thermostable polymerase, cloned into pTOPO-Zeroblunt (Invitrogen) and sequenced with the M13 forward (−20) primer. Sequence alignment with DNASIS-MAC v3.3 (Hitachi Solutions, Tokyo, Japan) allowed identification of changes from the parental sequences in each clone. The method for classification of gene conversion and non-templated point mutations was as previously described (33,34,35).

### Creation of the anti-POLD3 antibody

Chicken *POLD3* was amplified from DT40 cDNA with the primers 5′-ATA**GGATCC**ATGGAGGACGAGCTGTAC-3′ and 5′-GGC**GAATTC**TCATTTCTTCTGACAGAAG-3′ and cloned into pGEX-6P1 (Amersham, Buckinghamshire, UK) for expression as in GST-tagged form in *Escherichia coli* [BL21 STAR (DE3) (Invitrogen)]. Cells were induced with 1 mM Isopropyl β-D-1-thiogalactopyranoside (IPTG) and cultured for a further 4 h at 22°C. Cells were harvested, disrupted by sonication and the lysate cleared by ultracentrifugation. The lysate was passed over a glutathione sepharose 4B column and the GST-POLD3 protein eluted with 50 mM Tris–HCL pH 8.0, 150 mM NaCl, 20 mM glutathione. The eluted protein was then applied to a 1 ml HiTrap Q XL column (Amersham) and eluted with a NaCl gradient. GST-POLD3 eluted at 750 mM NaCl. For antibody production, two rabbits were immunized (Eurogentec, Seraing, Belgium) with 200 μg GST-POLD3. Serum was collected 4 weeks after the final immunization. The antibody was purified against a his-tagged POLD3 immobilized on an agarose column.

### Protein purification and primer extension assays

The human Polδ holoenzyme with or without POLD3, with N-terminal His-tagged p50, was expressed using a baculovirus vector (pBacPAK9, Clontech, Palo Alto, CA, USA) in insect cells (High Five, Life Technologies, Palo Alto, CA, USA), as described previously (36). The concentrations and purity of the proteins was estimated from the intensity of protein bands in an SDS-polyacrylamide gel (Supplementary Figure S6). For primer-extension analysis, DNA synthesis was carried out with 0.06 pmol ^32^P-labeled M17 primer (AGCTATGACCATGATTA) annealed with a 49-mer template oligo DNA (AGCTACCATGCCTGCACGAATXAAGCAATTCGTAATCATGGTCATAGCT), where X can be an abasic site. The assay was carried out in a reaction mixture (5 μl) containing 30 mM HEPES-NaOH (pH 7.4), 7 mM MgCl_2_, 8 mM NaCl, 0.5 mM dithiothreitol and 10 μM each dNTP in the presence of 2 nM of Polδ and 50 nM of PCNA for 15 min at 37°C. Used dNTP concentration reflects that in human cycling cells (37). At the end of the reaction, the products were denatured with formamide and loaded onto 15.6% polyacrylamide gels containing 7 M urea in Tris, Boric acid, EDTA (TBE) buffer (89 mM Tris, 89 mM boric acid, 2 mM EDTA). After electrophoresis, radioactivity was measured with a Fuji Image analyzer, FLA2500 (Fujifilm, Tokyo, Japan).

## RESULTS

### DT40 cells lacking POLD3 are viable but exhibit a moderate S phase delay

To explore the role of POLD3, we disrupted the *POLD3* gene in the chicken DT40 B cell line, creating *pold3* cells (Figure 1A and B). Loss of POLD3 expression was verified by RT-PCR and western blot analysis (Figure 1C and D). *pold3* cells are able to proliferate although their doubling time was 1.5× longer than that of wild-type cells (Figure 1E) due to a delay in late S-phase and a higher frequency of apoptosis (the left-most R5 gate, Figure 1F). To confirm this, we monitored cell cycle progression after release from synchronization in G_1_. Wild-type and *pold3* cells completed S-phase at 6 and 9 h after release respectively and returned to the next G_1_-phase at 9 and 12 h (Figure 1G). Thus, completion of DNA replication is delayed in the absence of POLD3.

### *pold3* cells have a normal velocity of DNA synthesis but an increase in unidirectional fork movement

To understand the nature of the S phase delay, we examined the kinetics of unperturbed DNA replication using molecular combing (Tables 1 and 2). Strikingly, the replication rate for wild-type and *pold3* cells in unperturbed conditions was not significantly different at 1.36 ± 0.05 kbp/min and 1.28 ± 0.03 kbp/min respectively (*P* > 0.34). The average inter-origin distance was also comparable between the two lines. These findings indicate that loss of POLD3 does not attenuate global progression of DNA replication forks on undamaged DNA templates (Table 1). However, *pold3* cells did exhibit an increase (20% as opposed to 13% in wild-type cells) in the proportion of unidirectional forks, in which one of the two forks heading away from a presumed origin is missing (Table 2, Supplementary Figure S1). Thus, POLD3 might be required for releasing replication forks that stall at spontaneously arising DNA damage on genomic DNA.

**Table 1.** Replication profiles of mutants in this study with and without UV treatment

Cell	UV	ΣIdU/ΣDNA (%)	Fork density (per Mb)	Average inter origins distances (kb)	Fork speed (kb/min)	Fiber length (kb)	Mb of DNA analyzed/number of fiber
Wild-type	−	47.05	8.63	81 ± 7	1.36 ± 0.05	368 ± 25	36.7/111
	+	44.21	8.00	76 ± 5	1.27 ± 0.06	360 ± 24	24.6/75
*pold3*	−	46.58	10.42	81 ± 4	1.28 ± 0.03	345 ± 17	62.4/190
	+	31.03	7.35	88 ± 7	1.14 ± 0.04	359 ± 23	28.8/89
*polζ/polη*	−	41.53	8.54	78 ± 5	1.40 ± 0.04	312 ± 14	42.9/153
	+	41.96	8.51	82 ± 4	1.24 ± 0.03	367 ± 28	48.2/149
*polζ/polη*	−	43.98	8.63	91 ± 8	1.40 ± 0.06	383 ± 24	21.9/58
*pold3/tet-ON*	+	46.18	8	78 ± 7	1.41 ± 0.08	326 ± 28	15.1/49
*polζ/polη*	−	49.57	6.94	85 ± 6	1.43 ± 0.06	341 ± 25	31.7/104
*pold3/tet-OFF*	+	53.27	8.32	73 ± 5	1.13 ± 0.05	304 ± 21	26.8/97

Profile of the DNA replication kinetics in indicated cells with or without UV irradiation was analysed by molecular combing.

**Table 2.** Proportion of unidirectional forks

Cell	Wild-type -UV	Wild-type +UV	*pold3* -UV	*pold3* +UV
Unidirectional fork%	13	18	20	26
Compared pair		*P*-values of chi-square test for pairwise comparison		
Wild-type − UV/Wild-type + UV		0.0206		
*pold3* − UV/*pold3* + UV		0.0201		
Wild-type − UV/*pold3* − UV		0.0006		
Wild-type + UV/*pold3* + UV		0.0116		

Proportion of unidirectional fork in indicated cells was examined as illustrated in Supplementary Figure S1.

### *pold3* cells exhibit sensitivity to a wide range of DNA damaging agents

**Figure 2.**
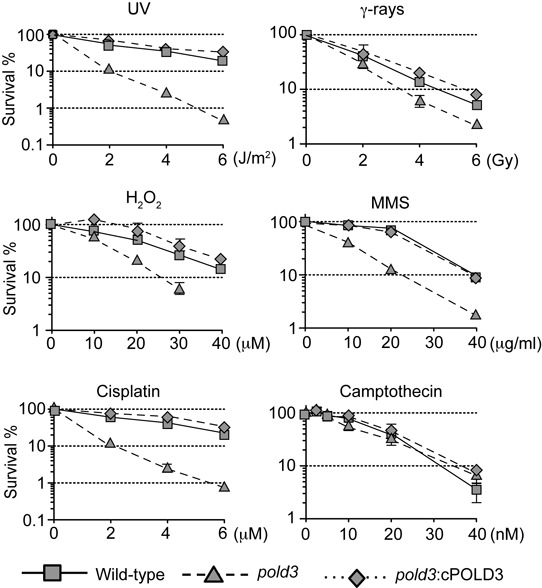
The POLD3 deficient cells are sensitive to a wide range of DNA-damaging agents. *pold3* cells exhibit hypersensitivity to various types of DNA damage. Cells with the indicated genotype were exposed to the indicated genotoxic agents. The dose of the genotoxic agent is displayed on the x-axis on a linear scale, while the percentage fraction of surviving cells is displayed on the y-axis on a logarithmic scale. Error bars show the SD of mean for three independent assays.

*pold3* cells exhibited sensitivity to a broad range of DNA damaging agents, including ionizing radiation (γ-rays), UV irradiation, the methylatingagent (methyl methane sulphonate, MMS), H_2_O_2_ and the chemical crosslinker (cisplatin) (Figure 2). This hypersensitivity to DNA damage was reversed by the ectopic expression of chicken POLD3 (Figure 2).

Since DNA polymerase δ and ζ are involved in a number of DNA repair and damage tolerance pathways (38), we next sought to define the pathways affected by loss of POLD3. The sensitivity of cells to the methylating agent, MMS and oxidising agent, H_2_O_2_ prompted us to test whether *pold3* cells exhibit a defect in base excision repair. However, gap filling during base-excision repair (39) occurred normally in *pold3* cells (Supplementary Figure S2). To investigate the involvement of POLD3 in nucleotide excision repair, we performed epistasis analysis of POLD3 and XPA, an essential protein of nucleotide excision repair (40). Inactivation of the *XPA* and *POLD3* genes had a synergistic impact on cellular sensitivity to UV, suggesting that POLD3 contributes to cellular tolerance of UV independently of nucleotide excision repair (Supplementary Figure S3). To investigate the involvement of POLD3 in HR, we measured cellular sensitivity to the topoisomerase I poison, camptothecin and gene targeting efficiency. *pold3* cells are not hypersensitive to camptothecin (Figure 2) and are proficient in gene targeting (Supplementary Table S1). Taken together, hypersensitivity of the *pold3* cells to various DNA damaging agents is not attributable to defects in the excision repair pathways or in HR.

### POLD3 is required for efficient Ig Vλ gene diversification by TLS

The wide-ranging hypersensitivity of *pold3* cells to DNA-damaging agents is reminiscent of the phenotype of TLS-deficient cells (41–43). To examine the role of POLD3 in TLS, we took advantage of the diversification of the Ig Vλ light chain locus of DT40 cells, described in the Introduction and summarized in Supplementary Figures S4 and S5.

**Figure 3.**
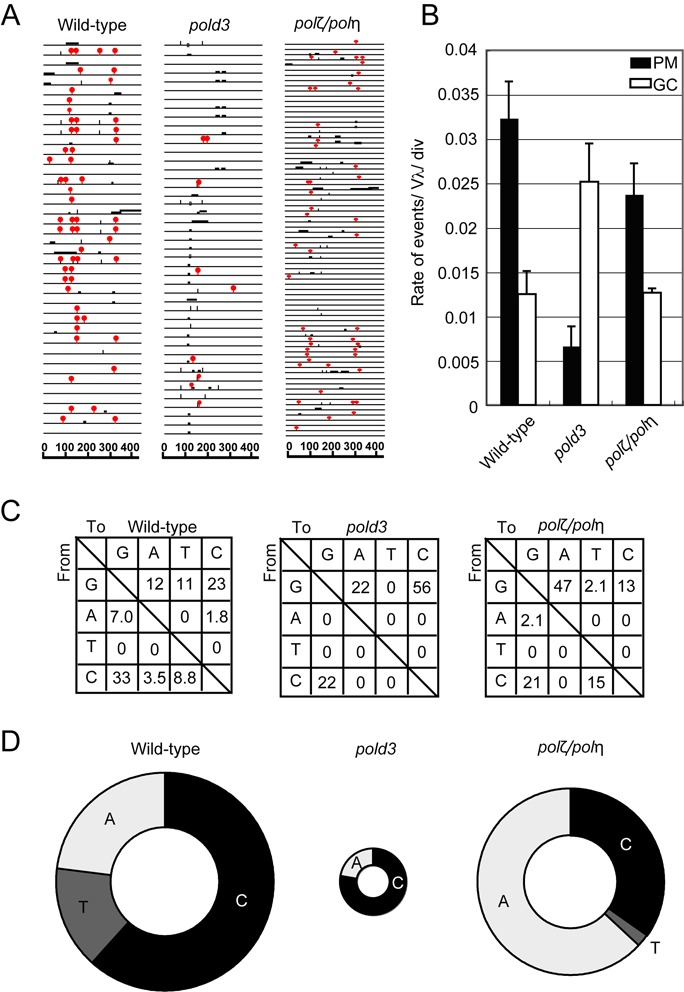
The important role of POLD3 in TLS past abasic sites during Ig V_λ_ hypermutation. (**A**) Ig Vλ segments isolated from indicated cells, clonally expanded for two weeks. Horizontal lines represent the rearranged Ig Vλ (450 bp), with hypermutation (red (gray in print) lollipop shapes), gene conversion (horizontal bars), single-nucleotide substitutions that could be the result of hypermutation or gene conversion (vertical bars) and single-base deletion (boxes) determined as described previously (34,35). More than three clones were expanded for two weeks and analyzed for each dataset. (**B**) The rates of gene conversion (GC) and hypermutation (PM) are indicated with standard error. (**C**) Pattern of point mutation in wild-type, *pold3* and *polζ/polη* cells. Tables showing the pattern of mutation in each line, given a percentage of mutations observed. (**D**) Frequency of mutagenic base insertion of C, T or A opposite C on either strand, corresponding to mutation from C to G, A and T, respectively. The size of the pie charts reflects the frequency of overall point mutation within mutated sequences, while the segments reflect the relative use of C, T or A in bypass.

To examine Ig Vλ diversification, we ectopically overexpressed AID and isolated at least three subclones each of wild-type and *pold3* cells (Supplementary Figure S4). Following two weeks clonal expansion, we compared the frequency and spectrum of mutations in the IgL variable segment in wild-type and *pold3* clones (Supplementary Figure S4). *pold3* cells exhibited a 4.5-fold reduction in the rate of non-templated mutations (Figure 3A and B). This reduced frequency of TLS was accompanied by a two-fold increase in the frequency of Ig gene conversion events suggesting that the failure of POLD3-dependent TLS results in more lesions being available to trigger Ig gene conversion and showing that POLD3 is not required for robust intragenic HR. We next looked in detail at the mutation spectrum of abasic site bypass. Under conditions of AID over-expression, 62% of the TLS in wild-type cells involves insertion of dC opposite the abasic sites created on both strands, leading to dC to dG transversions. This reaction is largely dependent on the deoxycytidyl transferase activity of REV1 (44,45). Following disruption of *POLD3*, the frequency of mutagenesis is decreased but 78% of the remaining mutations indicate insertion of dC opposite the abasic site. Intriguingly, few remaining mutations at dC in the *pold3* mutant appear to be highly strand biased. However, confirming this result will require a very much more substantial dataset.

Cells lacking Polζ alone do not tolerate AID overexpression, dying within a few days of retroviral infection (20). However, cells deficient in both Polη and Polζ are able to tolerate AID overexpression and exhibit a level of gene conversion and point mutagenesis comparable to wild-type cells, but with a marked increase in dA incorporation opposite the AID-induced abasic sites (20) (Figure 3). Thus, the immunoglobulin mutagenesis in cells lacking POLD3 and those lacking both Polη and Polζ is quite distinct. This suggests that while POLD3 contributes to TLS in the immunoglobulin loci, it does so substantially independently of Polζ. Moreover, *polζ* cells displayed significantly higher sensitivity to the ectopic AID expression in comparison with *pold3* cells, further suggesting functional independence of POLD3 and Polζ.

### Cells deficient in POLD3, Polη and Polζ are inviable

**Figure 4.**
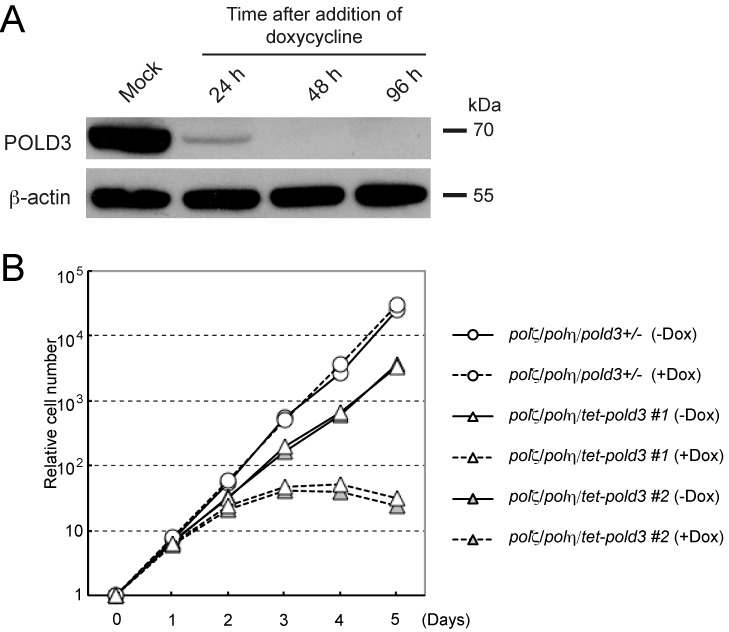
Synthetic lethality of *pold3* and *polζ/polη.* (**A**) Western blot for probing existence of POLD3 protein was shown. Protein samples from indicated cells were analyzed. β-actin was analyzed as a loading control. (**B**) Growth curves of the indicated cells. The transcription of *tet-POLD3* was active without doxycycline (−Dox) and inhibited upon addition of doxycycline (+Dox).

To further test whether POLD3 acts independently of the Polη/Polζ TLS axis, we conditionally disrupted POLD3 in *polη/polζ* double mutant cells by covering the deletion of POLD3 with a chicken *POLD3* transgene, the expression of which was under a tetracycline-repressible promoter (Figure 4A). *polη/polζ/pold3* triple mutant cells expressing the cPOLD3 transgene proliferated slightly more slowly than *polη/polζ/pold3^+/−^* cells (Figure 4B). Upon addition of doxycycline, POLD3 expression was significantly reduced by 24 h and lost completely by 48 h. By 3–4 days the cells had stopped proliferating. This observation further supports the idea that POLD3 plays a role that is independent of Polη and Polζ.

### POLD3 is required to maintain replication fork progression on UV damaged DNA but not to promote the filling of post-replicative gaps

**Figure 5.**
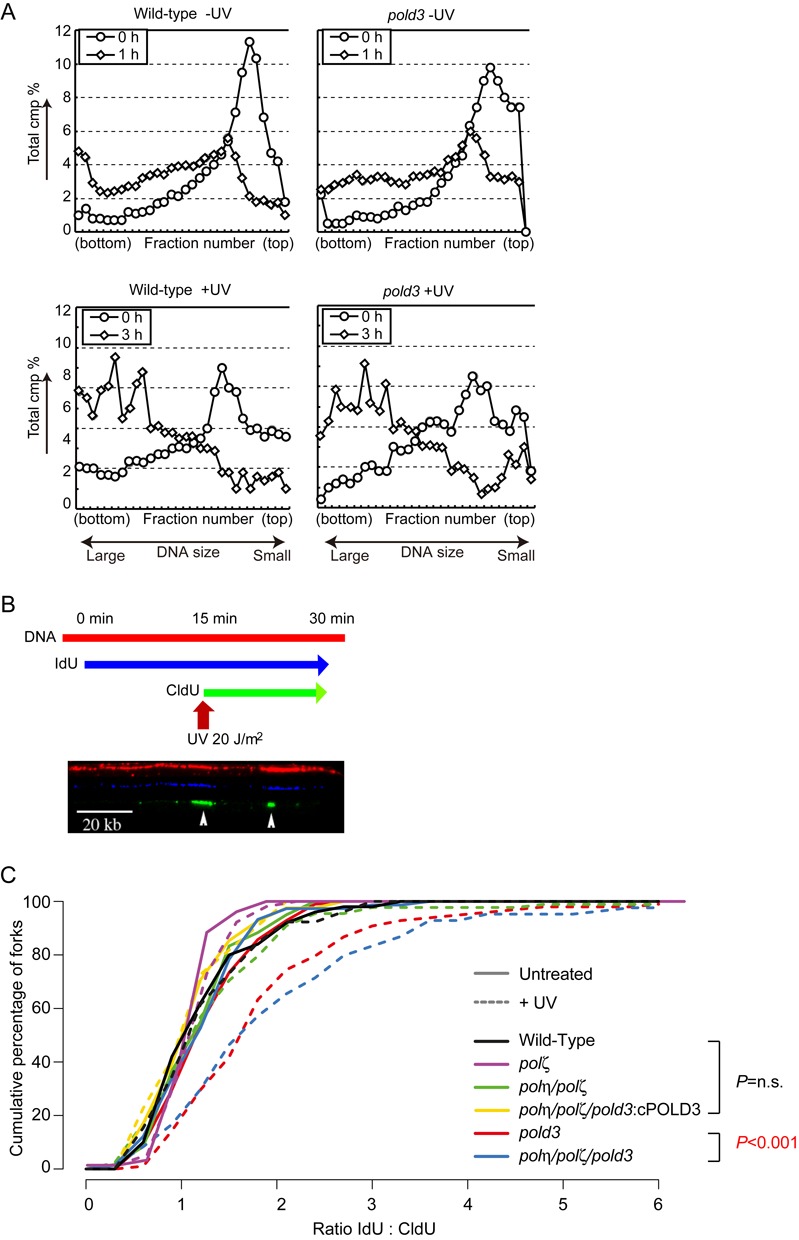
POLD3 but not Polζ is required to maintain replication fork progression on UV damaged DNA. (**A**) Intact postreplicative gap filling in *pold3* cells. Indicated cells without UV (upper panel) or with UV (5 J/m^2^) (lower panel) were pulse-labeled with [methyl-^14^C] thymidine, then incubated a further in fresh medium containing 10 μM unlabeled thymidine for the indicated time. Samples were separated on 5–20% alkaline sucrose gradient sedimentation. (**B**) Representative image showing stained DNA fibres. DT40 cells were labelled sequentially with IdU and CldU with or without UV treatment after IdU labeling. (**C**) The data for cells carrying the indicated genotypes was plotted as a cumulative percentage (y-axis) of forks at each ratio (x-axis). The *P*-values of the Kolmogorov–Smirnov test for ratio distribution of each mutant for UV compared to wild-type are indicated. n.s.: not significant.

Previous work in DT40 cells has revealed temporally separated modes of lesion bypass. One mechanism is responsible for timely filling of postreplicative gaps at UV-damage sites, while another operates at or very close to stalled replication forks and maintains normal fork progression on UV-damaged DNA (46). These modes of damage bypass can be assessed by alkaline sucrose gradient sedimentation of newly replicated DNA (Figure 5A) and DNA molecular combing (Tables 1 and 2, and Figure 5B and C), respectively.

We first asked whether POLD3 is required for post-replicative gap filling. Without exposure to UV, the ^14^C thymidine label was incorporated into higher molecular weight DNA similarly in wild-type and *pold3* cells (Figure 5A upper panel). This is consistent with the observation that POLD3 is not essential for unperturbed DNA replication (Table 1). However, *pold3* cells are also able to reconstitute high molecular weight DNA during a 3-h chase period following UV irradiation (Figure 5A lower panel) with similar kinetics to wild-type cells. Similarly, *polζ* cells exhibited reconstitution of higher molecular weight DNA following UV irradiation that was indistinguishable from wild-type cells (42). These data contrast with the defective postreplicative gap filling seen in *polη, rad18* and *pcna^K164R/K164R^* DT40 cells (46). Thus, neither Polζ nor POLD3 play a critical role in postreplicative gap filling at UV damaged sites in DT40.

We next examined replication fork progression after UV irradiation. To do this, we labeled nascent strands with IdU for 15 min, UV irradiated the cells and then continued labeling the nascent strands with CldU (Figure 5B and Table 1). After DNA combing, we revealed the tracts of CldU and IdU and calculated the ratio between them to allow comparison of the total DNA synthesized before and after UV exposure on a fork-by-fork basis. We plotted the data as a cumulative percentage of forks at each ratio (Figure 5C). *polζ* cells exhibit a response indistinguishable from wild-type i.e. a modest reduction in the average length of DNA synthesized during the second labeling period following UV (Figure 5C). This is consistent with a previous report examining Pol*ζ*-deficient mouse cells (47). In contrast, *pold3* cells exhibit a significant reduction in the length of DNA synthesized during the second labeling period after UV, consistent with a significantly increased proportion of forks stalling at UV lesions (*P* = 2.77 × 10^−7^, Supplementary Figure S6). In the time course of this experiment (15′ after 20 J/m^2^ UV), we estimate that forks would have about a 60% chance of encountering a UV dimer (based on (46), Supplementary Figure S1). Thus, POLD3 is required to maintain replication fork progression on a UV damaged template independently of Pol*ζ*. Taken together, these data indicate that POLD3 facilitates TLS independently of Pol*ζ*, leading us to speculate that it may facilitate TLS by Polδ itself.

### POLD3 facilitates *in vitro* TLS past abasic sites by Polδ

**Figure 6.**
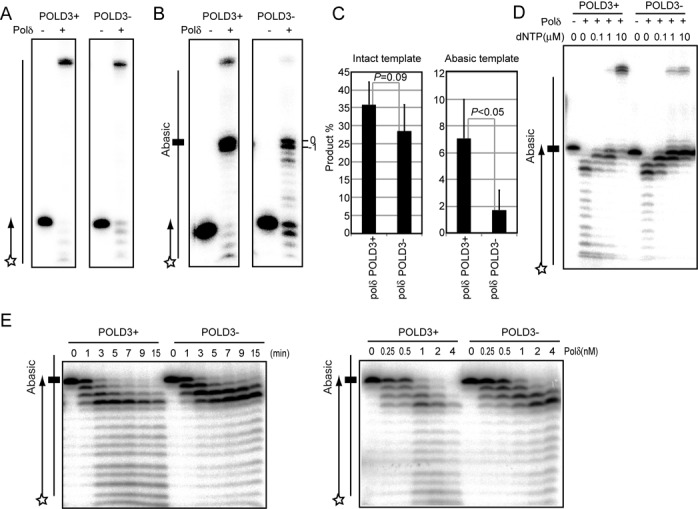
POLD3 is required for efficient Polδ-dependent TLS past abasic sites *in vitro*. (**A** and **B**) DNA synthesis reactions carried out with the indicated Polδ holoenzymes (2 nM each) on template and primer strands, which are schematically shown on the left. The 49-nucleotides template strands carry a dC (A) or abasic site (B) at the 25th nucleotide from the 3′ end. The 5′ end of the primer was ^32^P labeled as shown by a star. The DNA synthesis was done the presence of NaCl (8 mM), MgCl_2_ (7mM), PCNA (50 nM) and dNTP (10 μM), a concentration which reflects that in human cycling cells (37). After incubation at 37°C for 10 min, reaction products were separated on a 15.6% polyacrylamide gel containing 7 M urea and analyzed using a PhosphoImager. (B) The bands shown by −1 and +0 represent the DNA strand having stopped its extension one nucleotide before and at the abasic site, respectively. (**C**) The amounts of extension product (49 nucleotides) relative to the amount of input primer is shown as percentage. The experiment was performed at least three times, and averages are presented with SD and *P*-values. (**D**) Degradation and extension of the primer carrying a dA opposite the abasic site of the shown template DNA strand with the indicated Polδ holoenzymes (2 nM each) was examined at 15 min. The reaction was carried out with the indicated concentrations of dNTP. Less than 0.1 μm concentrations of dNTP significantly enhanced the proofreading exonuclease over polymerase activity. (**E**) Kinetics of the primer degradation in the absence of deoxynucleotides was examined. (Left) Reaction was carried out with 2 nM of the indicated Polδ holoenzymes for the indicated time. (Right) Reaction was carried out with the indicated concentrations of Polδ holoenzymes for 15 min.

To address the possibility that TLS by Polδ might be modulated by POLD3, we purified the intact human Polδ holoenzyme (Polδ (POLD3^+^)) and POLD3-deficient holoenzyme (Polδ (POLD3^−^)). We employed the human Polδ holoenzyme since the method for protein purification has been established (48). Polδ (POLD3^+^) and Polδ (POLD3^−^) were expressed and purified with the same efficiency (Supplementary Figure S7), indicating that the absence of POLD3 does not diminish the stability of the other three components of the holoenzyme. While the Proliferating cell nuclear antigen (PCNA) binding capability of POLD3 may increase the processivity of Polδ on a long circular template (49), to focus our analysis on the TLS capability of Polδ, we used a short (49-mer) linear template. As expected from the *in vivo* data shown in Table 1 and Figure 5A (upper panels), DNA synthesis by purified Polδ (POLD3^+^) and Polδ (POLD3^−^) on an intact template DNA strand was comparable (Figure 6A and C). However, bypass replication across an abasic site was significantly reduced in Polδ (POLD3^−^) (Figure 6B and C). The bypass is performed in two steps, the insertion of a nucleotide opposite to the lesion followed by extension of the resulting mismatch. Polδ (POLD3^+^) and Polδ (POLD3^−^) performed the insertion step with comparable efficiency, as the yield of product indicating an incorporation opposite the abasic site (‘0’ in Figure 6B) relative to that reflecting stalling at the −1 position was very similar with both Polδ (POLD3^−^) and Polδ (POLD3^+^). On the other hand, Polδ (POLD3^+^) was able to extend beyond the abasic site and complete DNA synthesis much more efficiently than Polδ (POLD3^−^) (Figure 6B and C). The reduced extension by Polδ (POLD3^−^) might be attributable to (i) higher exonuclease activity of Polδ (POLD3^−^) and/or to (ii) lower extension ability from the mismatched primer terminus than Polδ (POLD3^+^). To address these possibilities, we measured degradation and extension using a 28-mer primer, which carries dA in its 3′ terminus opposite the abasic site in template DNA. The ratio between intact and degraded primers in Polδ (POLD3^+^) and Polδ (POLD3^−^) was indistinguishable (Figure 6D). Moreover, the kinetics of the primer degradation in the absence of deoxynucleotides was also indistinguishable between Polδ (POLD3^+^) and Polδ (POLD3^−^) (Figure 6E). Thus, we propose that POLD3 is required for effective coupling of nucleotide insertion with subsequent extension thereby enhancing the overall processivity of Polδ during abasic site bypass.

## DISCUSSION

Here we have shown that a vertebrate cell line is capable of proliferating and replicating undamaged DNA with apparently normal kinetics in the absence of the POLD3 subunit of Polδ. This suggests that POLD3 does not play a major role in the central function of Polδ as the main lagging strand replicase (1). This rather surprising finding contrasts with the essential function of the POLD3/Pol32 ortholog of the fission yeast *S. pombe* (12). Further, while Polδ is involved in excision repair (2–4) and recombination (5–6,50), we could not detect any gross defects in these repair pathways in *pold3* cells. Rather, we found that POLD3 contributes significantly to cellular tolerance of a wide variety of DNA damaging agents by presumably promoting TLS.

It has been known for some time that Pol32 plays a role in TLS in *S. cerevisiae* and that genetically it played roles that are both dependent and independent of REV3, the catalytic subunit of Polζ, depending on the mutator examined (13–15,51–53). Recent biochemical studies have shown that Pol32 is not only a subunit of Polδ but is also an integral component of Polζ (16,17). This has led to the attractive suggestion that this association with Polζ explains the role of POLD3 in TLS and has led to the suggestion that Pol32 may facilitate switching between Polδ and Polζ (16–19). POLD3 and POLD2 are also subunits of human Polζ, interacting with the C terminal iron–sulphur clusters of REV3 in a similar manner to that reported in yeast (18,19). However, to date genetic data addressing the role played by vertebrate POLD3 in TLS have been lacking. The data we present here highlight striking phenotypic differences between cells lacking POLD3 and those lacking Polζ. Together with the lethality that results when both are disrupted the data suggest that the main role played by POLD3 in TLS in DT40 cells is independent of Polζ.

An important question is how POLD3 promotes TLS independently of Polζ. An obvious and attractive potential explanation is that POLD3 facilitates lesion bypass by Polδ itself. While Polδ is stalled by many lesions, there is good *in vitro* evidence that it is able to insert and extend over a number of single base lesions both with and without PCNA (48,54–56), including abasic sites (48,56–57). We show that purified human Polδ (POLD3^+^) is able to insert and extend over an abasic site at a physiological concentration of dNTP (Figure 6). Polδ preferentially inserts dA (57), an activity that could explain the bias toward the incorporation of dA we observed in the IgVλ gene in the absence of Polζ and Polη. How might POLD3 facilitate TLS by Polδ, given that it appears largely dispensable for general replication? We show that while Polδ (POLD3^+^) and Polδ (POLD3^−^) insert a single nucleotide opposite an abasic site with very similar efficiency, Polδ (POLD3^+^) is much more proficient at extension of the resulting mismatch (Figure 6B and C). This more efficient extension is attributable to higher processivity, but not lower proofreading exonuclease activity, in the presence of POLD3 (Figure 6D and E). It is not unlikely that Polδ (POLD3^+^) would cycle opposite the abasic site with nucleotide insertion balanced by exonucleolytic excision, with occasional extension events from the inserted nucleotide allowing for completion of the bypass of the abasic site. A possible explanation for the role of POLD3 would be that it helps prevent Polδ dissociating from the mismatched primer terminus, increasing the chance of a successful extension by POLD1.

Importantly, our data do not exclude cooperation between POLD3 and Polζ. Despite our data supporting Polδ being able to perform abasic site TLS on its own, independently of Polζ, it may remain the case that the efficiency of extension *in vivo* is facilitated by Polζ, which is able to complete bypass regardless of which polymerase mediated the insertion step. However, this role may become essential when the ability of Polδ to extend directly is compromised by loss of POLD3. Such a model would explain the synthetic lethality of cells lacking both POLD3 and Polζ and would suggest that the non-redundant function of the two proteins is in mediating the extension step of abasic site bypass.

Our study also indicates that POLD3 contributes to TLS operating at or close replication forks but not obviously to post-replicative gap filling. Previous studies both in DT40 cells (46) and mice (47) have shown that REV1 is also important for maintaining fork progression on damaged DNA and for effective Ig hypermutation (44,58). It is thus tempting to speculate that the two proteins may cooperate. Indeed, a physical interaction between Pol32 and REV1 has been described in *S. cerevisiae* (59), although this interaction has not been demonstrated in vertebrates. Nonetheless, it will be interesting to further explore the genetic and physical relationship between REV1 and POLD3 in vertebrates to examine whether POLD3 and REV1 collaborate to facilitate restart of replication by Polδ.

## SUPPLEMENTARY DATA

Supplementary Data are available at NAR Online.

SUPPLEMENTARY DATA
