# Supplementary material for: The POLD3 subunit of DNA polymerase δ can promote translesion synthesis independently of DNA polymerase ζ
Source: Nucleic Acids Res. 2015 Jan 27;43(3):1671–83. doi: 10.1093/nar/gkv023 (PMC4330384; doi:10.1093/nar/gkv023)
Supplement: SUPPLEMENTARY DATA [file supp_43_3_1671__index.html]

The POLD3 subunit of DNA polymerase δ can promote translesion synthesis independently of DNA polymerase ζ — SUPPLEMENTARY DATA 

# The POLD3 subunit of DNA polymerase δ can promote translesion synthesis independently of DNA polymerase ζ

## SUPPLEMENTARY DATA

**Files in this Data Supplement:**

- Supplementary Figures
- Supplementary Table
