# Supplementary material for: The POLD3 subunit of DNA polymerase δ can promote translesion synthesis independently of DNA polymerase ζ
Source: Nucleic Acids Res. 2015 Jan 27;43(3):1671–83. doi: 10.1093/nar/gkv023 (PMC4330384; doi:10.1093/nar/gkv023)
Supplement: SUPPLEMENTARY DATA [file supp_gkv023_nar-01798-d-2014-File008.pdf]

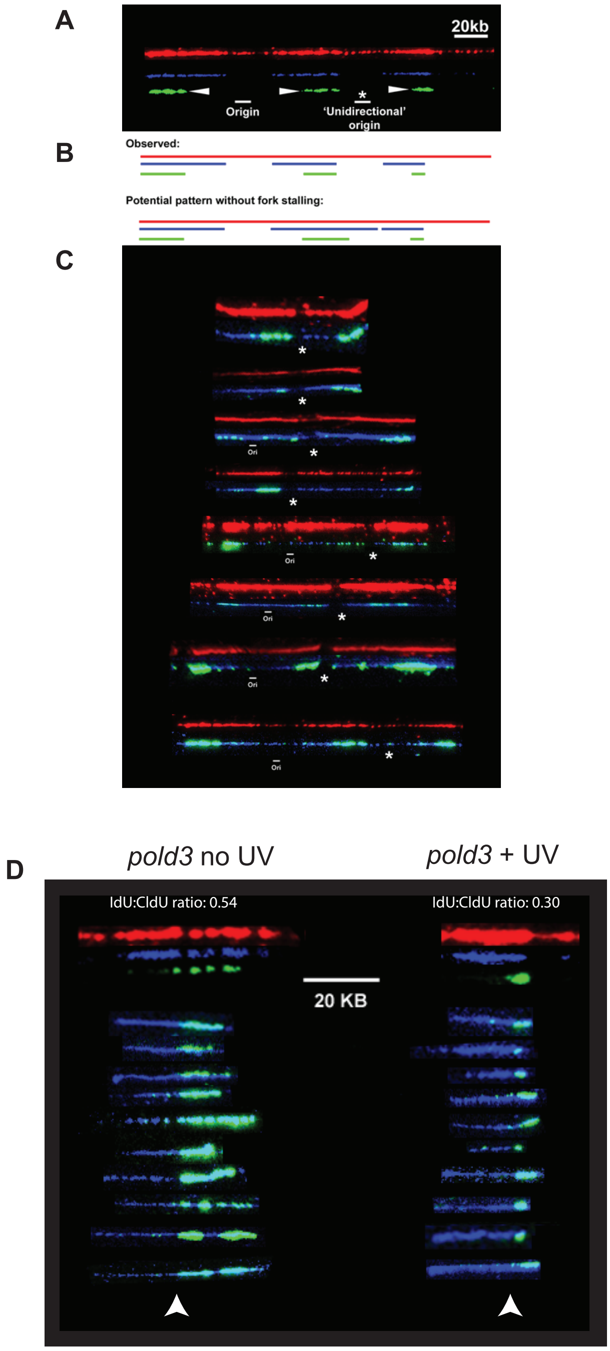

Figure S1. DNA combing: examples and analysis.

A - C. Analysis of unidirectional forks. Each replication origin that fires gives rise to two divergent replication forks. Thus, it should not be possible to have two consecutive forks traveling in the same direction. When those events occur, we assume that one of the two forks has collapsed. A. An example of a unidirectional fork. The three signals are separated: Red = DNA; Blue = IdU; Green = CldU. Arrows indicate the fork direction and an asterix indicates the vicinity of the putative fork collapse. B.Cartoon representing the corresponding fibres and what should have been seen in the absence of fork collpase. C. Further representative examples. D. Examples of replication arrest induced by UV. IdU is incoporated for 15 minutes followed by both IdU and CldU for another 15 minutes. During normal fork progression the IdU track should be twice as long as the CldU track. Thus the expected IdU:CldU ratio is 0.5. UV may be applied at the time of addition of the second label (indicated by the white arrow). These examples are from *pold3* cells. In order to avoid any truncated CldU tracks due to DNA fibre breakage, fibres whose DNA signal is longer than the replicative signals were examined (top example). For clarity the DNA signal is not shown in the additional examples.

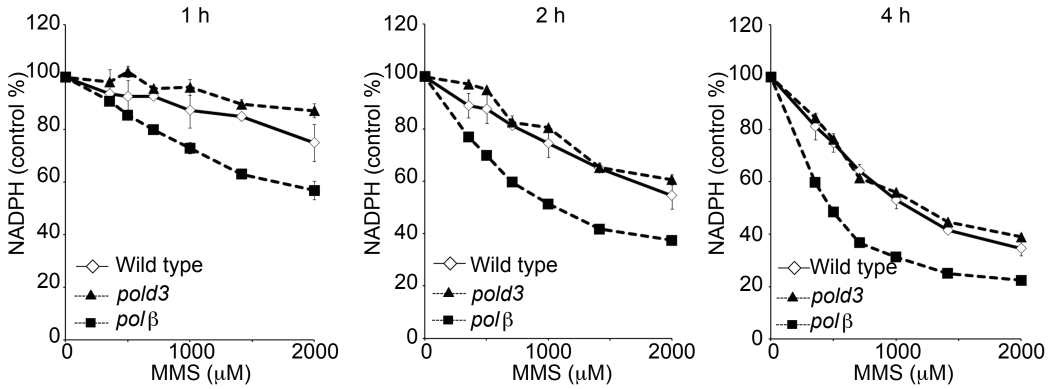

Figure S2 POLD3 contributes to MMS tolerance independent of base-excision repair pathway

To examine whether or not base excision repair is compromised in the *pold3* cells, we evaluated the gap-filling ability of the *pold3* cells in base excision repair. Since unrepaired single-strand gaps activate poly[ADP-ribose] polymerases and thereby cause a decrease in the amount of its substrate, NADPH, the gap-filling process was assessed by measuring the amount of NADPH. After treatment with MMS, NADPH was reduced in a dosage-dependent manner. Wild type (open diamond) and *pold3* cells (closed triangle) showed a similar reduction, while the polymerase β strain (*poll*β-/-, closed square) defective of base-excision repair showed a more pronounced reduction. Thus, base-excision repair is intact in the *pold3* cells.

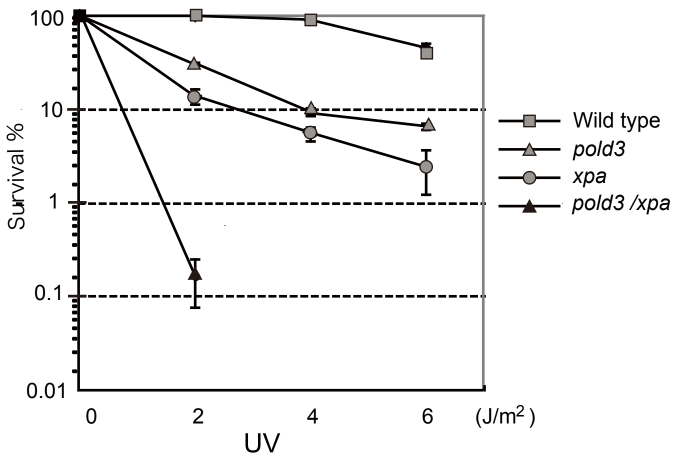

Figure S3 Genetic relation between *pold3* and *XPA*

To analyze the role of POLD3 in nucleotide-excision repair pathway, we disrupted the *XPA* gene, encoding a repair factor essential for nucleotide excision repair, in the *pold3*. A colony-formation assay was carried out to examine sensitivity to UV. Wild type, *pold3*, *xpa*, and *pold3*/*xpa* cells were exposed to UV (0, 2, 4, and 6 J/m2). The percent fraction of surviving colonies is displayed on the y-axis on a logarithmic scale. Error bars show the SD of mean for three independent assays. The loss of nucleotide excision repair increased the UV sensitivity of *pold3* cells to the same extent as in wild type cells, and critically a double mutant was much more sensitive indicating that POLD3 and nucleotide-excision repair contribute to UV tolerance independently of each other.

A

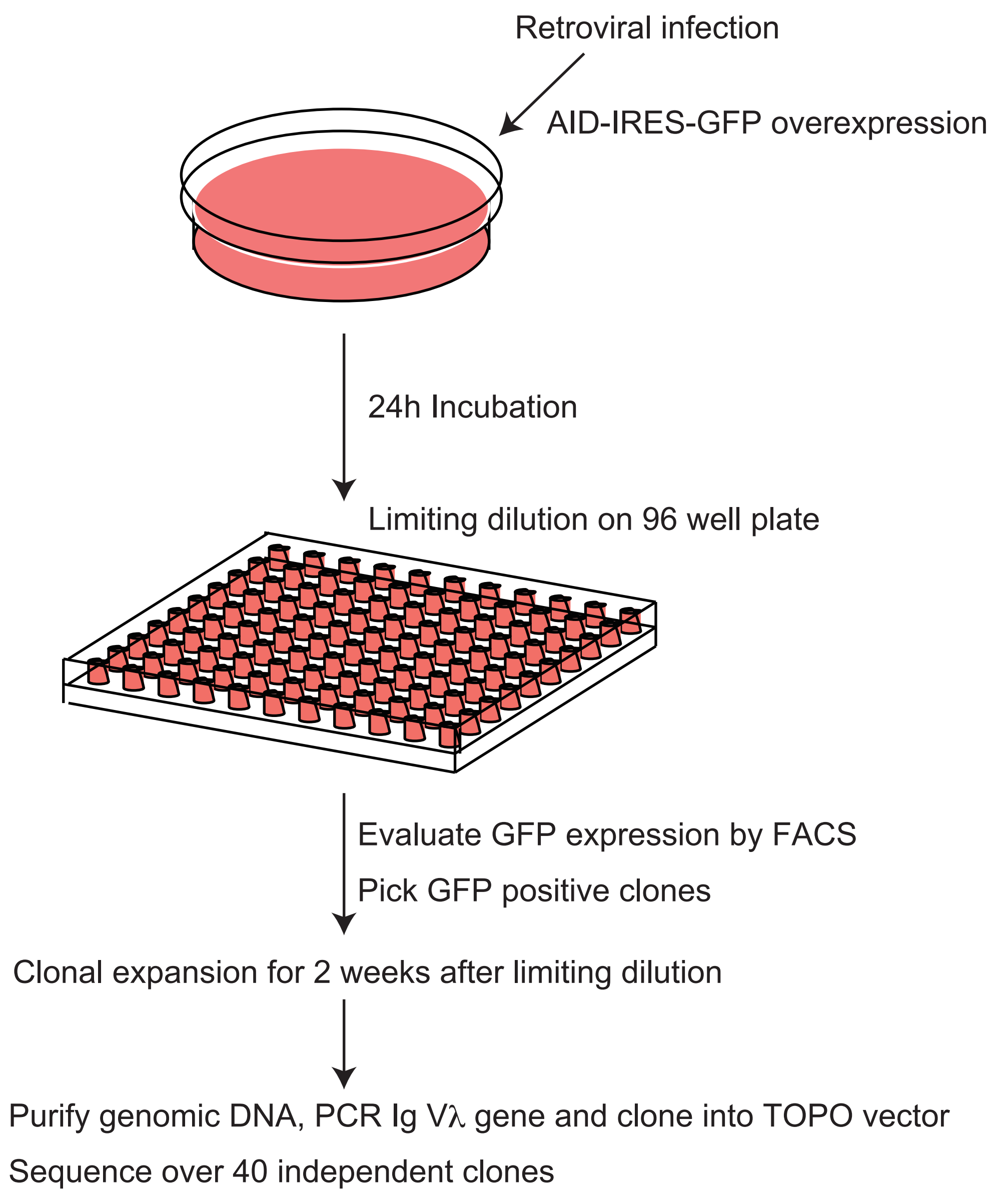

B

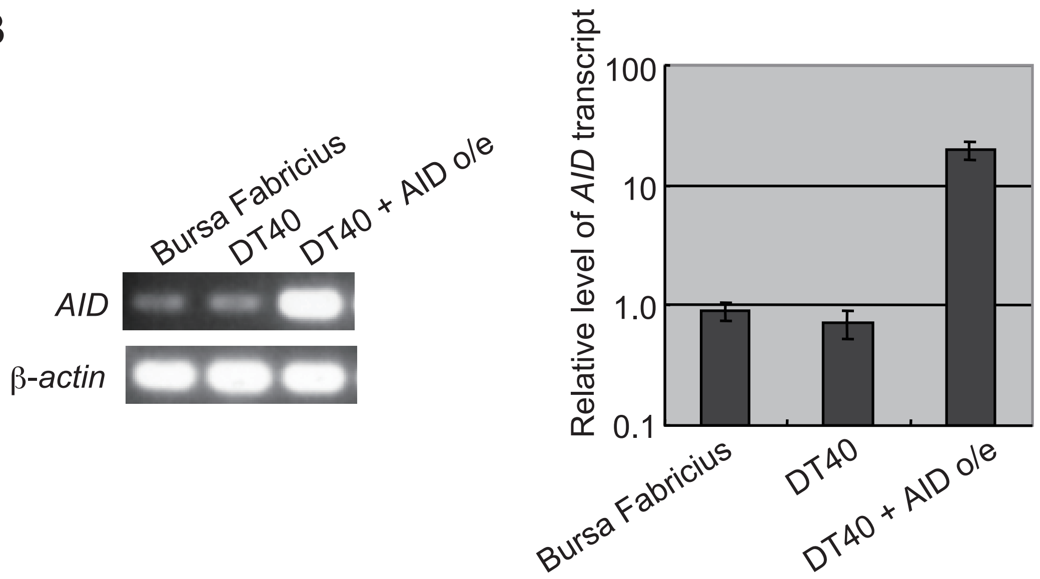

Figure S4 Schematic diagram of Ig Vλ gene diversification analysis

(A) The experimental procedure for Ig Vλ gene diversification analysis.
(B) Infection of AID expression virus into DT40 cells increases the level of *AID* by 20 fold.

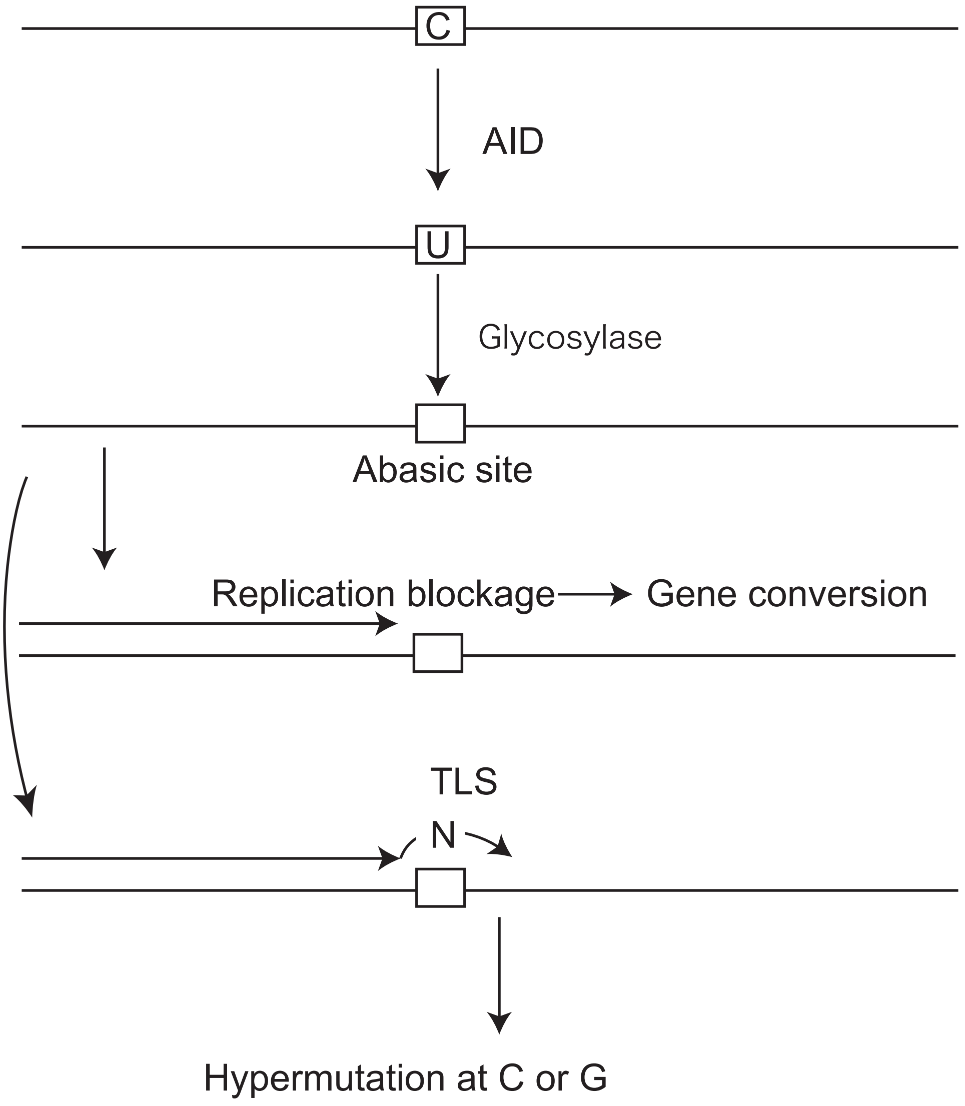

Figure S5 AID-dependent cytosine to uracil conversion initiates gene conversion and hypermutation in a chicken IgVλ segment.

AID deaminates cytosine and thereby converts it into uracil. This uracil base is removed by base-excision repair and abasic site is generated. Replication blockage at this site causes gene conversion. Translesion synthesis (TLS) causes hypermutation at the G/C pair.

IdU:CldU ratio distributions & estimation of stalled forks

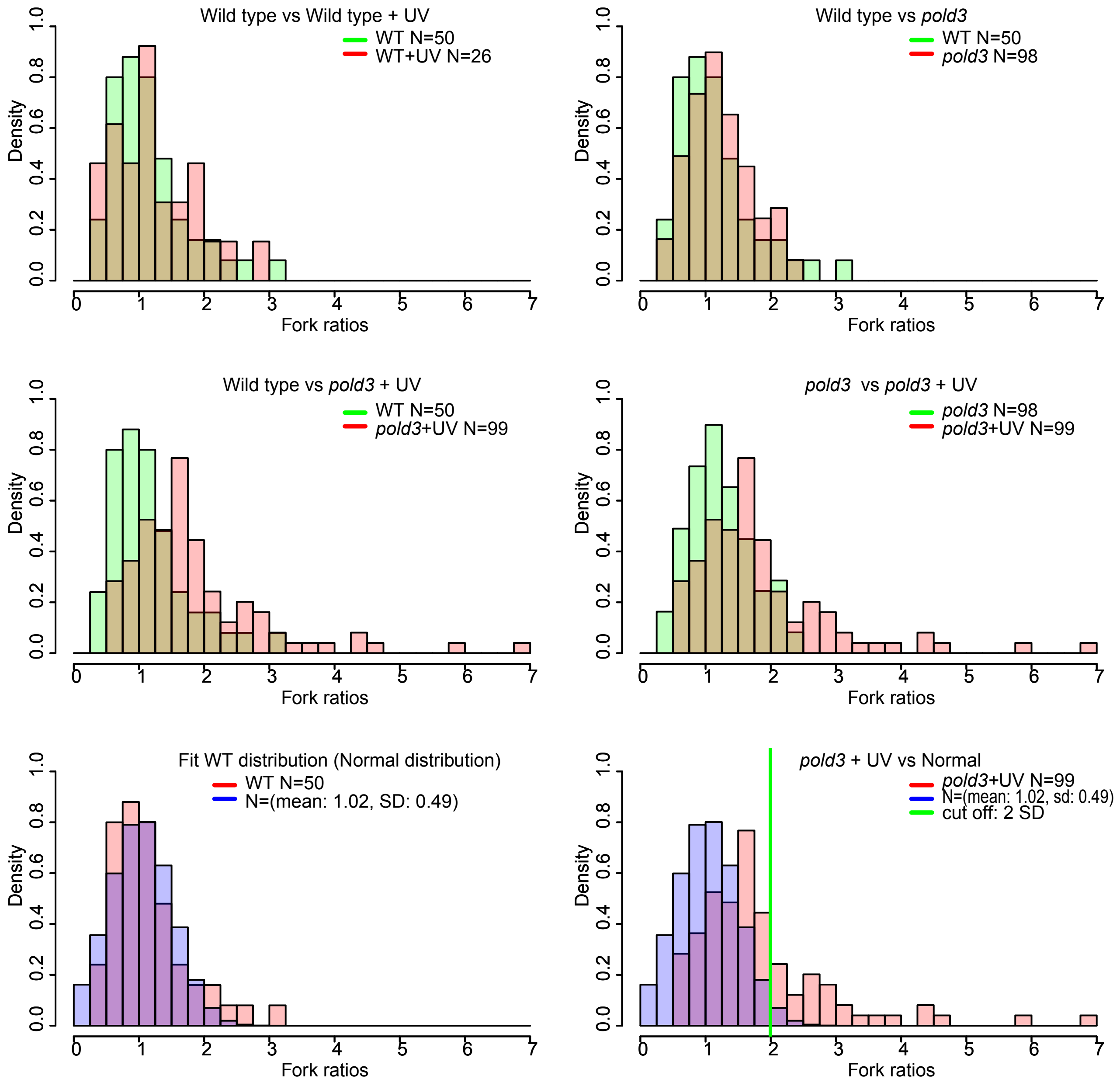

	WT	WT+UV	*pold3*	*pold3*+UV
# Forks observed	50	26	98	99
# forks > 2*SD	5	3	9	28
% forks > 2*SD	10	11.54	9.18	28.28
Binom test pValue / WT	1	0.74	1	2.77E-07

Figure S6 Replication fork stalling at UV lesion in *pold3* cells.

The IdU:CldU ratios in the indicated cells are presented as histogram. For unirradiated cells an IdU:CldU ratio of 1 suggests that the DNA synthesis is, on average, the same in the two labeling periods and that there is no fork stalling. There is a gaussian distribution around this average ratio. However, after UV in the *pold3* cells this gaussian shifts to the right and spreads out, consistent with an increase in stochastic shortening of the second label (i.e. fork stalling). If we take a threshold of for instance 2SD and ask what proportion of tracks have a ratio outside this range, we find the proportion of tracks rises from 10% in unirradiated cells (and, at this dose, irradiated wild type cells) to just over 28% ($p = 2.77 \times 10^{-7}$). This statistical analysis suggests an approximately 2.8-fold greater probability of stalling at a particular lesion in *pold3* than in wild type cells.

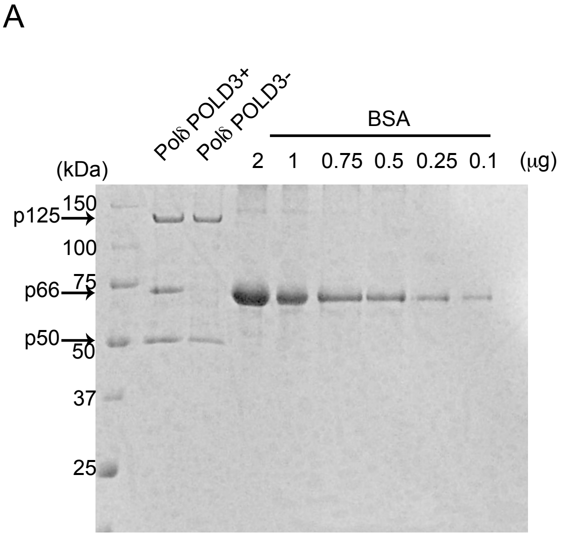

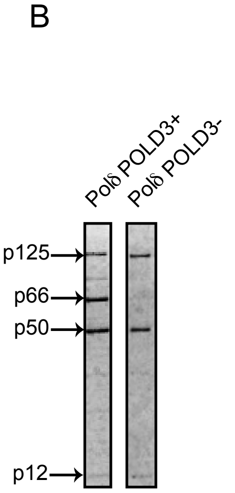

Figure S7 Purified human Polδ holoenzyme from insect cells.

(A-B) p50 was expressed in insect cells (High five, Invitrogen, USA) for Polδ (p125, p50, p66, and p12) and POLD3-deficient Polδ (p125, p50, and p12), with His-tagged N-terminal. The Polδ holoenzymes were purified in three steps. Polδ was initially purified by the affinity of the His-tag using a Ni-resin column (Probond, Invitrogen). Polδ was further purified by a MonoQ column with a linear increase of an NaCl concentration (0–500 mM). Finally, Polδ carrying a complete set of components was purified with glycerol gradient. Concentrations and purities of purified proteins were estimated from the intensities of the protein bands in an SDS-polyacrylamide gel using bovine-serum albumin as a standard. The yield of purified Polδ (POLD3-) holoenzyme was the same as that of Polδ (POLD3+), indicating that the loss of POLD3 did not affect the stability of the holoenzyme. (A) Coomassie Brilliant Blue staining of Polδ sample to estimate sample concentration. p12 subunit could not be detected in this gel condition due to small protein size. (B) Existence of the p12 subunit was confirmed by silver staining.
