# Supplementary material for: The POLD3 subunit of DNA polymerase δ can promote translesion synthesis independently of DNA polymerase ζ
Source: Nucleic Acids Res. 2015 Jan 27;43(3):1671–83. doi: 10.1093/nar/gkv023 (PMC4330384; doi:10.1093/nar/gkv023)
Supplement: SUPPLEMENTARY DATA [file supp_gkv023_nar-01798-d-2014-File009.doc]

Table S1 Gene targeting efficiency is preserved in *pold3* cells

| Cell | Targeting % |
| --- | --- |
| Wild type | 46% (22/48) |
| *Pold3* #1 | 60% (29/48) |
| *Pold3* #2 | 55% (26/47) |

To evaluate gene-targeting efficiency, we transfected CenpH-GFP knockin construct to indicated cells. The gene targeting event was examined by flow cytometry analysis.
